# Treatment of an external iliac artery chronic total occlusion using alternate access sites

**DOI:** 10.1186/s42155-019-0089-6

**Published:** 2019-12-13

**Authors:** Justin Ratcliffe, Mike Gorenchtein, Pankaj Khullar, Abel Casso Dominguez, Mohan Satish, Philip Green, Joseph Puma

**Affiliations:** 1 Department of Cardiovascular Medicine, Mount Sinai St. Luke’s Hospital, Icahn School of Medicine at Mount Sinai, New York, NY USA; 2 Department of Medicine, Mount Sinai St. Luke’s Hospital, Icahn School of Medicine at Mount Sinai, New York, NY USA; 3 Division of Cardiology, Department of Medicine, Columbia University Medical Center/New York-Presbyterian Hospital, New York, NY USA

**Keywords:** Chronic total occlusion, External iliac artery, Iliac artery stenting, Alternate access, Transpedal access, Peripheral arterial disease, Endovascular therapy

## Abstract

**Background:**

With the advent of endovascular techniques, alternate sites such as the pedal and radial arteries can now be accessed when treating peripheral arterial disease to reduce procedural complications, shorten recovery time, and improve patient comfort. However, a paucity of literature exists on the availability of support devices that can be utilized during challenging cases.

**Case presentation:**

A 70 year-old female patient presented for evaluation of severe lifestyle-limiting left-sided claudication refractory to maximal medical therapy. Angiography revealed a chronic total occlusion of the left external iliac artery, which was treated successfully by percutaneous intervention via a primary transpedal approach and with the assistance of the Outback® Elite re-entry device. The patient was discharged 2 h after the procedure and reported significant symptom improvement at follow-up.

**Conclusion:**

This case highlights a newly adopted endovascular approach through an alternate access site and illustrates how the Outback® Elite device can be used as an adjunctive tool in the treatment of complex lower-extremity vascular lesions.

## Background

Peripheral arterial disease (PAD) is a growing epidemic, affecting more than 8 million people in the United States (Allison et al. 2007). An endovascular approach, as opposed to standard surgical interventions, is becoming increasingly popular as the initial treatment option. Although transfemoral access has been traditionally selected for treating iliac artery lesions, alternative sites such as the pedal and radial arteries can be used to reduce vascular complication rates associated with femoral artery puncture (Kiemeneij et al. 1997; Vora and Rao 2014; Kwan et al. 2015). Recent studies have demonstrated the safety and procedural success of retrograde below-the-knee punctures, including the subintimal arterial flossing with anterograde-retrograde intervention (SAFARI) and the controlled antegrade retrograde reentry technique (CART), in peripheral endovascular interventions (Chou et al. 2016; Shishehbor and Jaff 2016; Ruzsa et al. 2018; Welling et al. 2018). A frequent concern with the transpedal approach is the perceived lack of availability of support devices during challenging cases. We describe the use of the Outback® Elite re-entry device in a case of a successful intervention of an external iliac artery chronic total occlusion (CTO) via a transpedal approach.

## Case presentation

A 70 year-old female with a prior history of a right carotid artery stenosis status post carotid endartectomy, hypertension, and dyslipidemia presented with severe lifestyle-limiting left-sided claudication (Rutherford class 3). The ankle-brachial index (ABI) was 0.60 and 1.02 on the left and right lower extremities, respectively. The patient’s symptoms persisted despite conservative therapy which included supervised exercise, aspirin, atorvastatin, and cilostazol. Following discussion of possible treatment options, including vascular bypass, a collective decision was made to proceed with a diagnostic peripheral angiogram and percutaneous endovascular intervention, if needed.

**Fig. 1:**
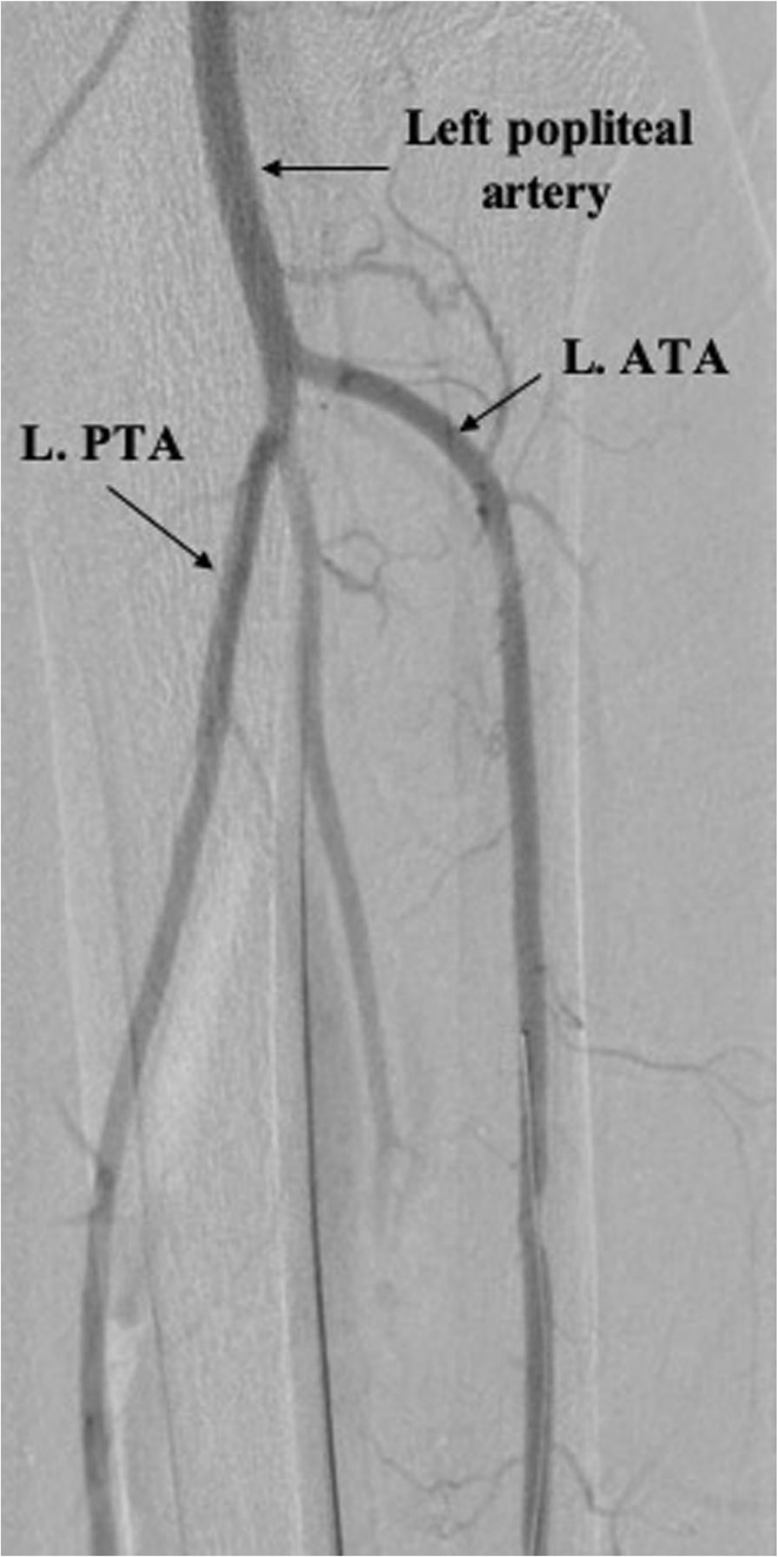
A tibial artery angiogram showing patency of the distal left popliteal artery, left anterior tibial artery (L. ATA), and the left posterior tibial artery (L. PTA)

**Fig. 2:**
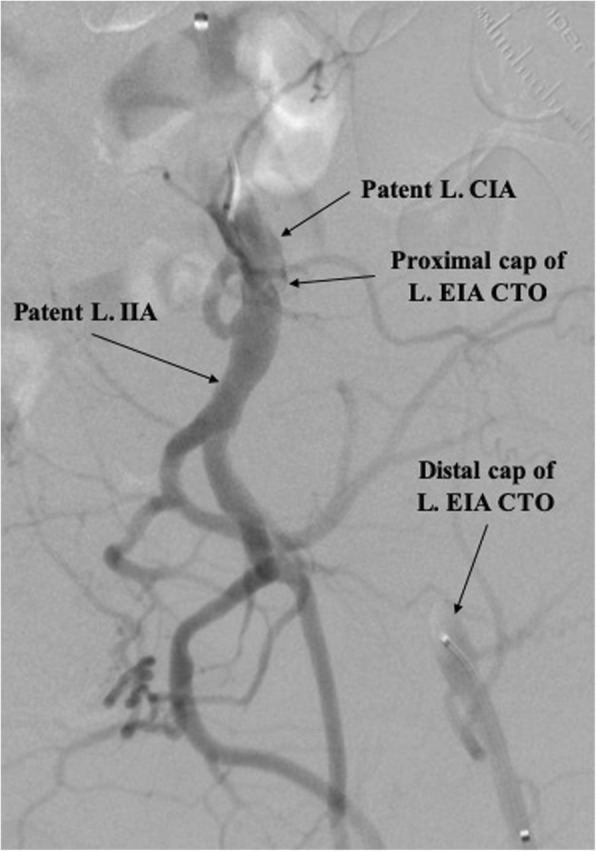
An angiogram from dual injections demonstrating the proximal and distal caps of a chronic total occlusion (CTO) throughout the entire length of the left external iliac artery (L. EIA). (L. CIA, left common iliac artery; L. IIA, left internal iliac artery)

The patient was brought to the catheterization laboratory and prepped in a sterile fashion. Ultrasound was used to confirm patency of the left dorsalis pedis artery (DPA) and anterior tibial (ATA) and guide cannulation of the DPA with a 21-gauge echogenic needle, followed by introduction of a 4-Fr Pinnacle Precision Glidesheath (Terumo) into the ATA. After confirming flow, nitroglycerin 200 mcg, verapamil 1 mg, and heparin 5000 IU were administered intra-arterially. To achieve an activated clotting time (ACT) > 300 s, additional heparin was given if needed. A tibial angiogram was performed to assess size and disease burden of the anterior tibial artery (Fig. 1). A 125 cm, 4-Fr Tempo Aqua Vertebral catheter (Cordis) was advanced over a 0.014-in., 300 cm Runthrough wire (Terumo) to perform a diagnostic angiogram of the left lower extremity, which showed the distal cap of a CTO of the distal left external iliac artery (EIA). To localize the proximal cap of the stenotic lesion, left radial access was obtained and a 4-Fr Multicurve 150 cm catheter (Terumo) was advanced into the left common iliac artery (CIA). Dual injections revealed CTO throughout the entire length of the left EIA (Fig. 2).

**Fig. 3:**
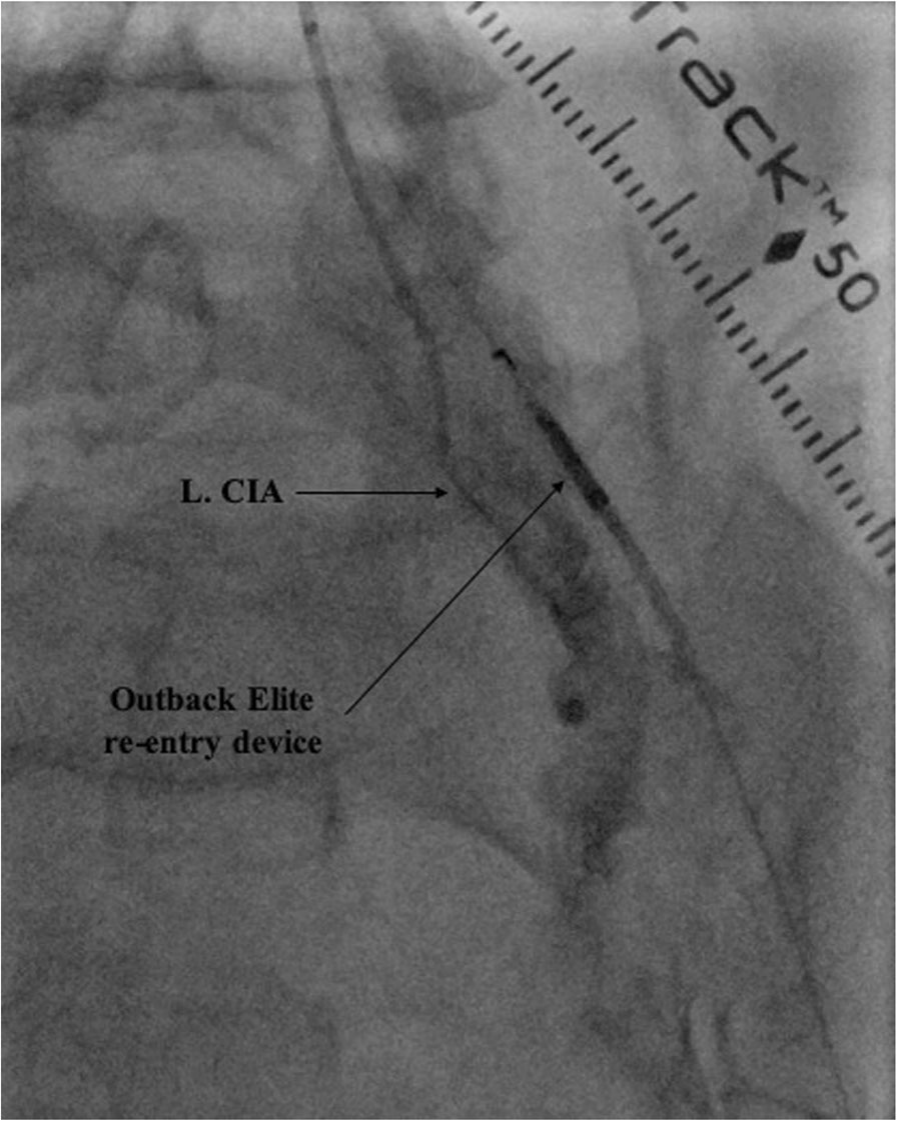
An angiogram showing advancement of the Outback Elite re-entry device (Cordis) into the dissection plane adjacent to the left common iliac artery (L. CIA)

**Fig. 4:**
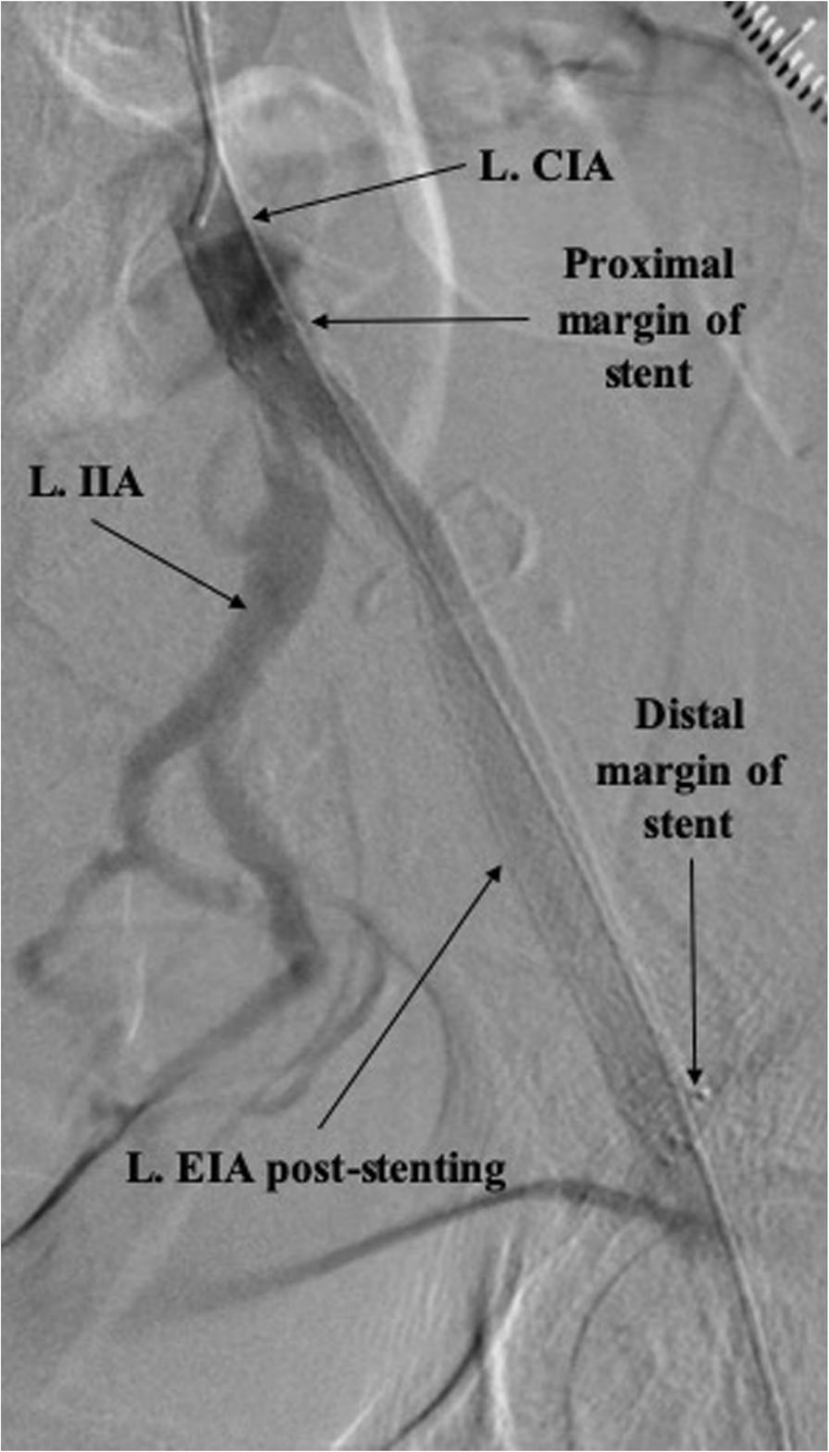
Post-intervention angiogram showing a patent left external iliac artery (L. EIA) stent as well as the proximal and distal margins of the stent. (L. CIA, left common iliac artery; L. IIA, left internal iliac artery)

The transpedal 4-Fr Glidesheath was upsized to a 6-Fr Slender Glidesheath (Terumo) in anticipation for retrograde intervention given the extent and complexity of the CTO. This Slender Glidesheath has an outside diameter equivalent to a 5-Fr sheath, but can accommodate 6-Fr equipment. We initially attempted antegrade recanalization, however, we were unsuccessful in breaking the proximal cap likely due to lack of support from radial access. Next, retrograde wire escalation attempts were made with a 0.018-in. Glidewire Gold (Terumo) and 0.035-in. Glidewire Advantage (Terumo) with entry into a dissection plane close to the true lumen of the CIA. In order to refrain from direct entry into the aorta and compromise the contralateral limb, the decision was made to directly reenter into the CIA. The wire was exchanged with a hydrophobic 0.014-in. Granslam wire (ASAHI) and an Outback Elite re-entry device (Cordis) was advanced into the dissection plane adjacent to the CIA (Fig. 3). Successful reentry was achieved and the wire was exchanged with a 0.035-in. Three hundred centimeter Supracore (Abbott), followed by balloon dilation of the stenosis and deployment of an 8 mm x100mm Absolute Pro self-expanding stent (Abbott) with excellent post-intervention results and retained patency of the ostial internal iliac artery (Fig. 4). Post-intervention tibial angiogram did not reveal any damage to the vessel access site and showed good patency of the DPA and ATA. The left radial artery and left DPA introducer sheaths were removed without complications. A TR Band (Terumo) was placed on the left radial artery puncture site and a VasoStat (Forge Medical, Inc.) was placed on the left DPA puncture site to achieve hemostasis. Once the Vasostat was placed over the DPA a vascular ultrasound was used distally to ensure vessel patency. The patient was discharged home 2 h after the procedure on atorvastatin 80 mg daily, and dual antiplatelet therapy with daily aspirin 81 mg and clopidogrel 75 mg for at least 1 month. Detailed clinical follow-up was performed at 6 and 12 months after procedure, during which the patient reported significant symptom improvement (Rutherford class 1) and had normal repeat left ABI and duplex ultrasound.

## Discussion

Historically, the femoral artery has been the original access site when treating PAD via percutaneous endovascular intervention (Shah et al. 2019). The transpedal approach was initially reserved as an adjunct or bailout for cases where femoral access has failed. However, recent reports have shown its feasibility and safety in both, the routine treatment of PAD and the treatment of complex vascular lesions (Amoroso et al. 2015; Chou et al. 2016; Shishehbor and Jaff 2016; Ruzsa et al. 2018). As shown from studies in cardiac catheterization, opting for alternative puncture sites has been significantly beneficial in reducing bleeding, hematomas, and pseudoaneurysms (Agostoni et al. 2004; Romagnoli et al. 2012; Ferrante et al. 2016). The other benefits of alternate access include patient comfort, quicker recovery time, shorter hospital stay, and overall reduction of economic burden on the healthcare system. In comparison to femoral access, transpedal route in particular has the potential benefit of reduced radiation and contrast doses and shorter fluoroscopy time, mainly due to the ease of advancing catheters and wires in retrograde fashion and the relative ease of crossing CTO lesions (Shah et al. 2019). Adequate patency of the distal tibial vessels is required procedures performed through transpedal access, which can be screened for with an arterial ultrasound.

In a case series described by Mustafa et al., PAD revascularization was performed in 23 patients via a primary tibio-pedal artery access without observed complications(Mustapha et al. 2014). These results were validated in a larger case series by Kwan et al., where a primary transpedal approach was selected for endovascular interventions, yielding favorable outcomes (Kwan et al. 2015). Similar success was achieved in superficial femoral artery interventions using the pedal artery as the sole access site(Amoroso et al. 2015). In the treatment of iliac artery disease specifically, successful interventions via a single transpedal access or dual transpedal and transradial access have also been recently described (Auguste et al. 2015; Zachariah et al. 2016).

One of the concerns that many operators have in adopting the pedal approach is the availability of support equipment for complex cases. In this case, we illustrate that the Outback re-entry device can be easily employed for high complexity lesions, such as an external iliac artery CTO, when wire escalation and dissection techniques are insufficient. This device is compatible with 6-Fr sheaths commonly used during transpedal access and is designed exclusively for use in the peripheral vasculature. Increased availability and diversity of dedicated support devices such as the Outback re-entry device will enhance procedure safety and efficacy. We equally acknowledge that procedural success is operator-dependent and may not be reproducible by operators who are not familiar with specific equipment, puncture techniques, and patent hemostasis methods. In addition, severe outflow disease in the superficial femoral artery or tibial vessels would need to be treated first before advancing the Outback device or stents to the level of the iliac arteries. Finally, given the risk of perforation and the potential need for covered stents when treating iliac artery stenosis, the contralateral groin was also prepped in case of bailout. A 7-Fr slender sheath was on standby for the pedal access in case a Viabahn stent was needed.

## Conclusion

This case report demonstrates the treatment of an external iliac artery CTO via the transpedal approach with the aid of the Outback**®** Elite Re-Entry Device. Given the potential benefits in avoiding femoral artery access, larger studies are needed to compare the different endovascular approaches including the scope of transpedal approach.

## Data Availability

Data sharing not applicable to this article as no datasets were generated or analyzed during the current study.

## Abbreviations

ABI: Ankle-brachial index
ACT: Activated clotting time
ATA: Anterior tibial artery
CIA: Common iliac artery
CTO: Chronic total occlusion
DPA: Dorsalis pedis artery
EIA: External iliac artery
IIA: Internal iliac artery
PAD: Peripheral arterial disease
PTA: Posterior tibial artery
